# Immunohistochemical study for the expression of leukocyte adhesion molecules, and FGF23 and ACE2 in *P. gingivalis* LPS-induced diabetic nephropathy

**DOI:** 10.1186/s12882-020-02203-y

**Published:** 2021-01-06

**Authors:** Koichiro Kajiwara, Yoshihiko Sawa, Takahiro Fujita, Sachio Tamaoki

**Affiliations:** 1 Department of Oral Growth & Development, Fukuoka Dental College, 2-15-1 Tamura, Sawara-ku, Fukuoka, 814-0193 Japan; 2 Department of Oral Function & Anatomy, Okayama University Graduate School of Medicine, Dentistry and Pharmaceutical Sciences, 2-5-1 Shikata-cho, Kita-ku, Okayama, 700-0914 Japan

**Keywords:** *P. gingivalis*, LPS, Diabetic nephropathy, VCAM-1, E-selectin, ACE2, FGF23

## Abstract

**Objective:**

The present study aims to examine the expression of leukocyte adhesion molecules and renal metabolic factors in diabetic mouse kidneys with periodontal pathogen Pg-LPS-induced nephropathy.

**Background:**

We recently reported that the glomerular endothelium expresses toll-like receptor (TLR)2 and TLR4 in diabetic environments and TLR2/4 ligand *Porphyromonas* (*P.*) *gingivalis* lipopolysaccharides (Pg-LPS) induce nephropathy in diabetic mice. It is thought that Pg-LPS promotes the chronic inflammation with the overexpression of leukocyte adhesion molecules and renal-specific metabolic enzymes by the recognition of Pg-LPS via TLR in the diabetic kidneys. There have been no reports of the effects of periodontopathic bacteria on the expression of leukocyte adhesion molecules and the accumulation of physiologically active substances in the kidney.

**Methods:**

The immunohistochemical investigation was performed on diabetic mouse kidney with Pg-LPS-induced nephropathy with glomerulosclerosis in glomeruli.

**Results:**

The**r**e were no vessels which expressed vascular cell adhesion molecule-1 (VCAM-1), E-selectin, or fibroblast growth factor (FGF) 23 in streptozotocin (STZ)-induced diabetic ICR mice (STZ-ICR), or in healthy ICR mice administered Pg-LPS (LPS-ICR). However, in diabetic ICR mouse kidneys with Pg-LPS-induced nephropathy (LPS-STZ) the expression of VCAM-1 and the accumulation of FGF23 were observed in renal tubules and glomeruli, and the expression of E-selectin was observed in renal parenchyma and glomeruli. The angiotensin-converting enzyme 2 (ACE2) was detected in the proximal tubules but not in other regions of ICR, STZ-ICR, or LPS-ICR. In LPS-STZ ACE2 was detected both in renal tubules as well as in glomeruli. The Mac-1 and podoplanin-positive cells increased in the renal parenchyma with diabetic condition and there was the distribution of a large number of Mac-1-positive cells in LPS-STZ.

**Conclusions:**

The Pg-LPS may induce diabetic renal inflammation such as glomerulosclerosis and tubulitis with infiltration of Mac-1/podoplanin positive macrophages via glomerular overexpression of VCAM-1 and E-selectin, resulting in accumulation of both ACE2 and FGF23 which were unmetabolized with the inflammation-induced kidney damage under the diabetic condition. Periodontitis may be a critical factor in the progress of nephropathy in diabetic patients.

## Background

Diabetic nephropathy is a serious complication in diabetes mellitus, caused by glomerulosclerosis, with renal failure arising from dysfunction of glomerular capillaries. Critical factors in diabetic nephropathy have been thought to be advanced glycation end products (AGE) and hydroxyl radicals which induce oxidative stress and the production of various cytokines through the recognition of AGE in a hyperglycemic environment [1–3]. However, the factors which cause individual differences in the development of nephropathy in diabetic patients are not well elucidated. Hyperglycemia induces the expression of TLR2 and TLR4 through PKC-α and PKC-δ, respectively, with the stimulation of NADPH oxidase in monocytes [4, 5]. The renal metabolic recognition of AGE by not only AGE receptor but also toll-like receptor (TLR) has been suggested as one candidate for the occurrence of diabetic nephropathy [6–8]. The TLR is a sensor for bacterial components like lipopolysaccharide (LPS) and high levels of expression of TLR2 and TLR4 has been reported in blood cells of diabetic nephropathy patients [9–13]. It has been established that the TLR ligand engagement induces the production of inflammatory cytokines as well as leukocyte adhesion molecules, which activate renal inflammation causing glomerulosclerosis [14–18]. There are also reports that the periodontal pathogen *Porphyromonas* (*P.*) *gingivalis* becomes a risk factor in cerebrovascular diseases and atherosclerosis [19, 20]. Lipopolysaccharides (LPS) are produced in the outer membrane of *P. gingivalis* and act as not only a periodontal pathogen leading to periodontal tissue destruction but also a risk factor in cardiovascular disorders [21, 22]. It is well studied that *P. gingivalis* LPS (Pg-LPS) is recognized by host defense systems via TLR4 to Pg-LPS lipid A and via TLR2 through co-purifying molecules in the Pg-LPS prep in LPS-accumulated organs and induces the expression of leukocyte adhesion molecules [8, 23–25].

The expression of TLR2/TLR4, and adhesion molecules go up in kidneys of diabetic mice [8, 11, 26]. We recently reported that the glomerular endothelium of streptozotocin (STZ)-induced diabetic mice expresses TLR2 and TLR4 genes and proteins in glomeruli [27], and that all Pg-LPS-administered diabetic mice reached the humane endpoint during the period in which all of the diabetic mice without the LPS administration and Pg-LPS-administered non-diabetic mice lived without any symptoms [28]. In diabetic mice Pg-LPS promoted the production of urinary protein and glomerulosclerosis with the accumulation of type 1 collagen and inflammatory cytokines in glomeruli. Further, the progress of diabetic nephropathy was suppressed in TLR4 blockage Eritoran-administered diabetic mice [29]. Since the severe periodontitis causes bacteremia, it is thought that microorganisms of the oral cavity enter the renal circulation of patients with severe periodontal disease through the systemic circulation. It appears that Pg-LPS accumulated in glomeruli may induce chronic renal inflammation as a result of the leukocyte migration.

The abnormal and overexpression of leukocyte adhesion molecules, renal metabolic enzymes, and physiologically active substance has been reported in the kidneys of diabetic and other renal autoimmune diseases, such as erythematosus and IgA nephropathy. However, there have been no reports of the effects of periodontopathic bacteria on the expression of leukocyte adhesion molecules and the accumulation of physiologically active substances in the kidney. The overexpression of vascular cell adhesion molecule-1 (VCAM-1) is observed in the renal proximal tubules in renal immune diseases with tubulitis by acute renal allograft rejection [30–34]. The overexpression of E-selectin is observed on intertubular capillaries in glomerulonephritis [35, 36]. Osteocyte-derived hormone fibroblast growth factor (FGF) 23 acts as a key regulator of the renal phosphate metabolism which reduces renal phosphate uptake. In chronic kidney disease serum FGF23 levels are massively elevated [37–39]. Angiotensin-converting enzyme 2 (ACE2), a monocarboxypeptidase, that cleaves a typical renal pressor hormone angiotensin (Ang) II into Ang 1–7 and degrades Ang I to Ang 1–9, displays antihypertensive and organ-protective effects. The ACE2 is usually observed in proximal tubular epithelial cells but increases in diabetic kidney and hypertensive renal diseases [40, 41]. Considering these findings, the overexpression of leukocyte adhesion molecules and accumulation of unmetabolized renal-specific hormone, and enzymes may occur in diabetic kidneys by the renal inflammation associated with TLR recognition of *P. gingivalis*. The present study aims to examine the expression of leukocyte adhesion molecules and FGF23, and ACE2 in the mouse kidney with Pg-LPS-induced diabetic nephropathy.

## Methods

### Animals

The animal study was conducted to investigate the expression of VCAM-1, E-selection, FGF23, and ACE2 in Pg-LPS-induced diabetic nephropathy. The animal use protocol of the experiments was approved by the Animal Experiment Committee of Fukuoka Dental College (No. 19010). The study in the present report used 4 groups (non-treated control, LPS-administered non-diabetic control, diabetic control, LPS-administered diabetic experimental) with 6 mice in each group. We decided the animal number according to the decision by the Animal Experiment Committee of Fukuoka Dental College based on the appropriate number of animals in biomedical research from the viewpoint of animal welfare [42]. The number of animals that can set the probability of α error to an appropriate low level is 5 in general animal studies. So, in the case of genetically identical animal groups of the same lineage, 5 or more animals per group, and the test using 4 groups including the control group, are considered appropriate. All experimental specimens were harvested from euthanized mice and the experimental protocol followed ARRIVE guidelines. The 4-week-old male mice of the ICR closed line were purchased from a commercial vendor (Kyudo, Fukuoka, Japan). Animal upkeep and experiments were performed in the Fukuoka Dental College Animal Center under the following conditions and procedures described elsewhere [29]: normal feeding in a 100% controlled atmosphere which had passed an examination for bacteria in a room where the temperature and humidity were completely controlled. The health status and humane endpoints of the mice were assessed daily and mice which had lost the ability to ambulate and to access food or water were euthanized.

Anesthesia and euthanasia were conducted in compliance with the AVMA guidelines for the euthanasia of animals 2020 Edition and with the methods in the explanation of standards for rearing and storage of experimental animals and relief of pain, which was established by the Study Group on Standards for Animal Breeding and Storage in the Japanese Ministry of the Environment. Namely, euthanasia was performed by induction anesthesia (1 l/min of 2% isoflurane mixed with 30% oxygen and 70% nitrous oxide with an anaesthetic apparatus) followed by intraperitoneal injection with sodium pentobarbital (150 mg/kg, Sumitomo Dainippon Pharma Co., Ltd., Japan) and cervical dislocation. For drug administration inhalation anesthesia with 5% concentration isoflurane was performed to mice placed in an anesthesia box with careful observation. For blood sampling inhalation anesthesia or abdominal anesthesia was performed. Namely, in inhalation anesthesia isoflurane was initially introduced at a concentration of 4–5% and then maintained at about 2–3%. The abdominal anesthesia was performed by intraperitoneal administration of anesthetic and sedative mixture: medetomidine chloride (0.3 mg/kg, Meiji Seika Pharma Co., Ltd., Tokyo, Japan), midazolam (4 mg/kg, Maruishi Pharmaceutical Co., Ltd., Osaka, Japan), and butorphanol tartrate (5 mg/kg, Meiji Seika).

The STZ-injected ICR mice were used as a diabetic model and STZ and Pg-LPS injected ICR mice were used as a Pg-LPS-induced diabetic nephropathy model. Mice were given a single intraperitoneal injection of STZ (Sigma-Aldrich Japan, Tokyo, Japan) and the blood glucose of mice were checked by a Glutest Sensor (Sanwa Kagaku Kenkyusyo CO., LTD., Nagoya, Japan) twice a week after the injection. The STZ-injected ICR mice which showed extremely elevated blood glucose levels of over 600 mg/dl were used as STZ-induced diabetic mice (STZ-ICR). The Pg-LPS of 3 mg/kg (LD50 = 30 mg/kg body weight; Invivogen, San Diego, California, USA) which had been confirmed to have no effect on the health condition in healthy ICR in our previous study was consecutively injected just below the buccal mucosa of ICR once a week for 4 months. Mice were monitored for sugar, protein, and bleeding in urine by urine reagent strips (Uriace, Terumo Corporation, Tokyo, Japan), and the blood which collected from the tail vein under anesthesia was analyzed for blood urea nitrogen (BUN) and creatinine (CRE) by Kyudo Co., LTD (Tosu, Japan). When mice showed strongly positive for sugar and protein in urine by the reagent strips, and simultaneously showed the BUN levels of above 40 mg/dl and the CRE levels of above 0.7 mg/dl, they were used as STZ and Pg-LPS-induced diabetic nephropathy mice (LPS-STZ) according to our previous study [29]. In summary, the present study was performed with 24 ICR mice divided into four groups (*n* = 6 per group) which is the smallest unit to achieve reliable statistical processing: groups of mice without any treatment (healthy control, ICR), ICR with only LPS treatment (experimental infection model, LPS-ICR), ICR with only STZ treatment (experimental diabetes model, STZ-ICR), and ICR with LPS and STZ treatment (experimental diabetic nephropathy model, LPS-STZ). All mice in the experimental groups were maintained by careful observation every day and were euthanized at the end of the designated period of the experiments, and tissue from the mice was collected.

### Immunohistochemistry

The present study performed the investigation by immunohistochemistry following the method described elsewhere [29]. Briefly, frozen mouse kidney tissue sections were fixed in 100% methanol and treated with primary antibodies (1 μg/ml): hamster monoclonal anti-mouse podoplanin clone 8.8.1 (#127402, BioLegend Inc., San Diego, CA, USA) as a well-known podocyte and macrophage marker, rat monoclonal anti-mouse E-selectin/CD62E (#112734, R&D Systems Inc., Minneapolis, MN, USA), rat monoclonal anti-mouse VCAM-1/CD106 (#96419, R&D Systems), rabbit polyclonal anti-mouse FGF23 (#bs-5768R, Bioss Inc., Boston, MA, USA), rabbit polyclonal ACE2 (#ab15348, Abcam plc., Cambridge, UK), and rat polyclonal anti-mouse CD11b/macrophage-1/Mac-1 clone M1/70 (#557394, BD Biosciences, San Jose, CA, USA). After the treatment the sections were exposed with secondary antibodies (0.5 μg/ml): Alexa Fluor 488 or 568-conjugated goat anti-hamster (#A21110, Thermo Fisher Scientific Inc., Molecular Probes Invitrogen, Eugene, OR), goat anti-rabbit (#A11077, Thermo), or goat anti-rat IgGs (A11011, Thermo). The immunostained sections were examined by microscope digital camera systems with a CFI Plan Apo Lambda lens series and DS-Ri2/Qi2 (Nikon Corp., Tokyo, Japan). All experiments were replicated several times [5–10] with different sections.

### Measurements of immunostained area

Immunostained areas were captured under the same conditions and measured in 20 area of 4 sections for each immunostaining in microscopic images at 200x magnification by ImageJ (National Institutes of Health, Bethesda, MD) as described elsewhere [27–29]. The values were obtained by sum of the surface areas of pixel regions recognized by ImageJ in the sections unresponsive to the secondary antibody alone. The relative expression area reacted with primary antibodies was expressed by arbitrary units of the ratio: positive area of antibody reactions in the sections of STZ-ICR and LPS-STZ / the area of LPS-ICR.

### Statistics

All experiments were repeated five times, and data are expressed as the mean + SD. The statistical significance of differences (*P* < 0.01) was determined by one-way ANOVA and the two-tailed unpaired Student’s *t* test with STATVIEW 4.51 software (Abacus concepts, Calabasas, CA, USA). Mean values were calculated with standard deviation (STDEV). The corresponding author is fully aware of the group allocation at the different stages of the experiments. The data analysis and assessments were performed by all co-authors.

## Results

### Immunostaining of adhesion molecules and leukocytes

**Fig. 1:**
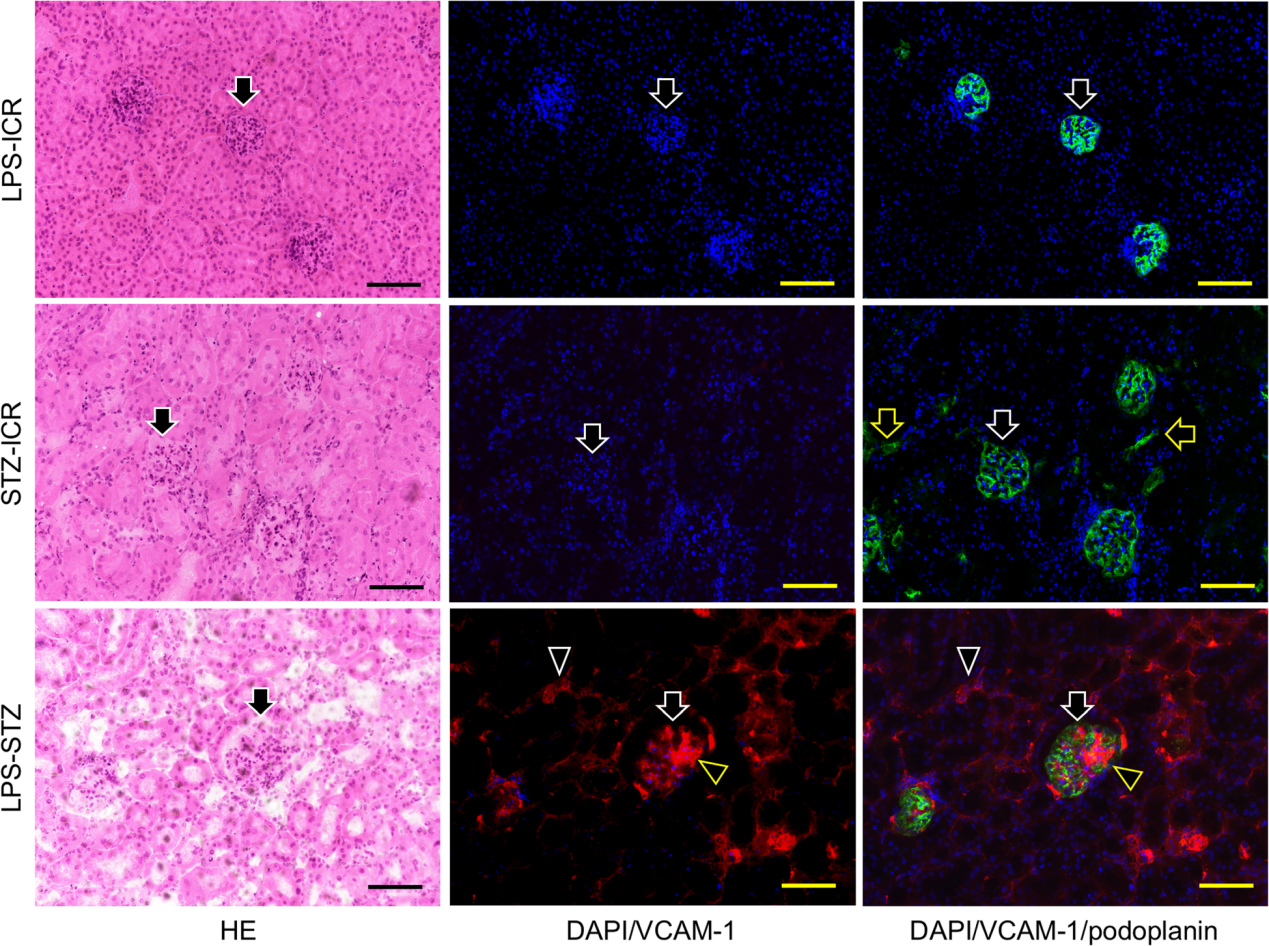
Immunostaining of VCAM-1 in the diabetic mouse kidney with Pg-LPS-induced nephropathy. Hematoxylin-Eosin staining (HE) (left column); immunostaining for VCAM-1 (red) (center column); and merged immunostaining for VCAM-1 and podoplanin (green) (right column), with DAPI staining of nuclei (blue). The glomerular epithelial cells were immunostained by anti-podoplanin to be able to discriminate glomeruli (arrows). Reaction with anti-VCAM-1 were not identified in LPS-ICR (top row) or in STZ-ICR (middle row) while the reaction was identified in renal tubules (arrowheads) and in glomeruli (yellow arrowheads) LPS-STZ (bottom row). Podoplanin-positive macrophages were also identified in STZ-ICR (yellow arrows). Bars: 100 μm

**Fig. 2:**
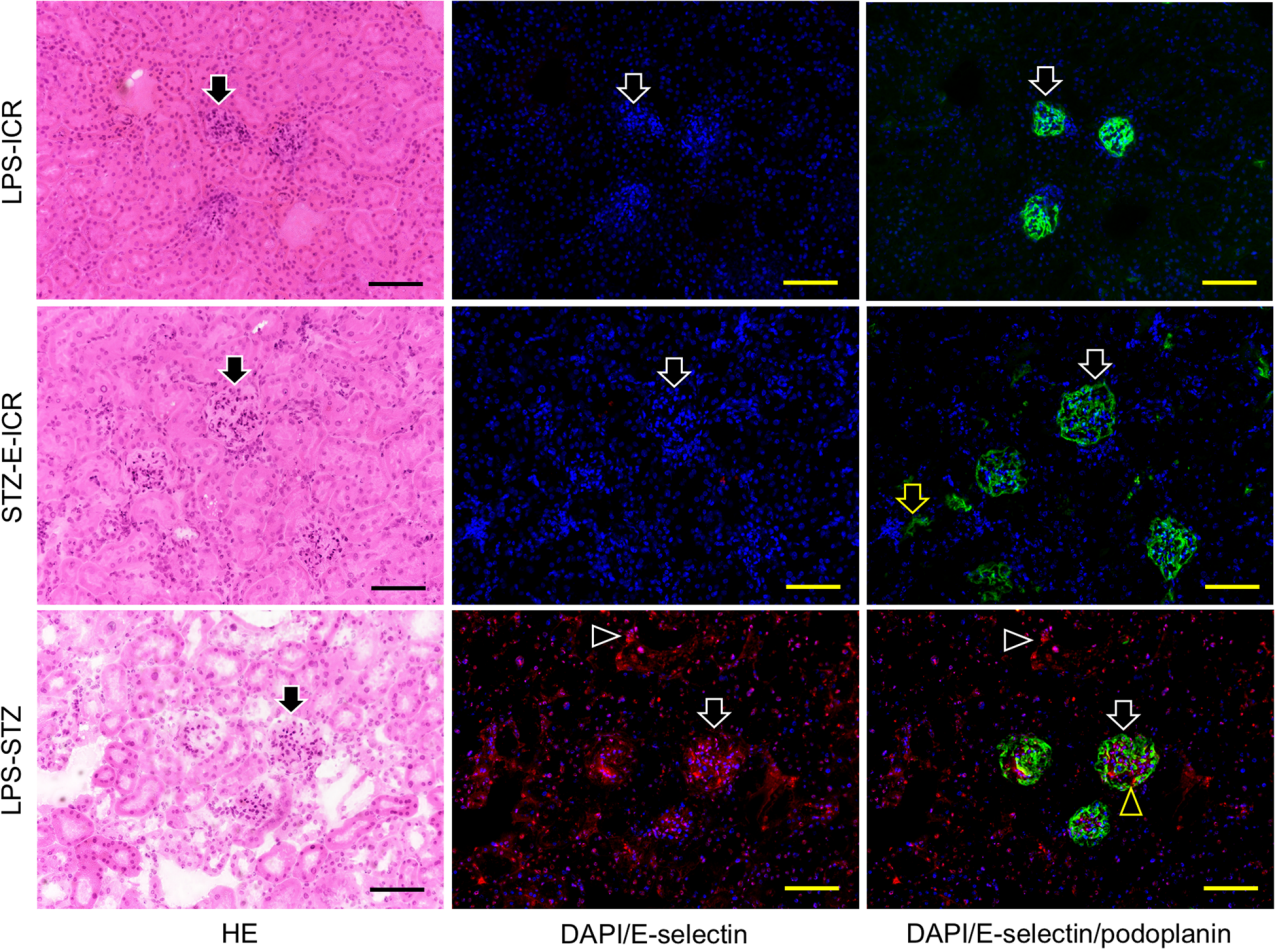
Immunostaining of E-selectin in the diabetic mouse kidney with Pg*-*LPS-induced nephropathy. Hematoxylin-Eosin staining (HE) (left column); immunostaining for E-selectin (red) (center column); and merged immunostaining for E-selectin and podoplanin (green) (right column), with DAPI staining of nuclei (blue). The glomerular epithelial cells were immunostained by anti-podoplanin to discriminate glomeruli (arrows). Reaction with anti-E-selectin were not identified in LPS-ICR (top row) or STZ-ICR (middle row) while the reaction was identified in the whole renal parenchyma (arrowheads) including glomeruli (yellow arrowheads) in LPS-STZ (bottom row). Podoplanin-positive macrophages were also identified in diabetic mouse kidneys (yellow arrow). Bars: 100 μm

**Fig. 3:**
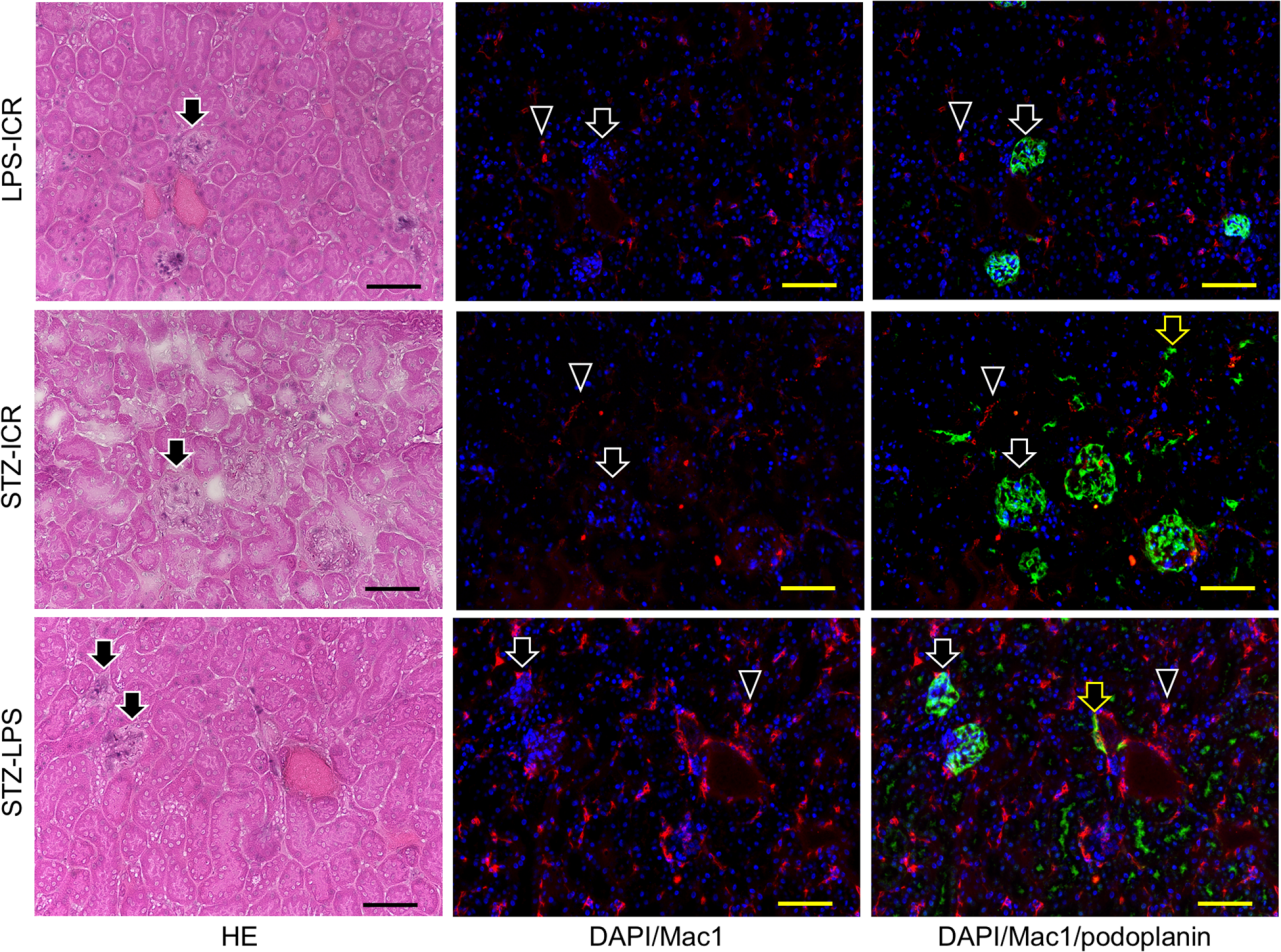
Immunostaining of Mac-1 in the diabetic mouse kidney with Pg*-*LPS-induced nephropathy. Hematoxylin-Eosin staining (HE) (left column), immunostaining for Mac-1 (red) (center column), and merged immunostaining for Mac-1 and podoplanin (green) (right column), with DAPI staining of nuclei (blue). The glomerular epithelial cells are immunostained by anti-podoplanin to be able to discriminate glomeruli (arrows). Mac-1-positive cells (arrowheads) were identified in the whole renal parenchyma (arrowheads) of LPS-ICR (top row) and STZ-ICR (middle row) at a similar level while the number of Mac-1-positive cells increased in LPS-STZ (bottom row). Podoplanin-positive macrophages were also identified in diabetic mouse kidneys (yellow arrows). Bars: 100 μm

Immunoreaction products with anti-VCAM-1 were not detected in kidneys of ICR (Supplementary 1), LPS-ICR or STZ-ICR while the products were identified in renal tubules and glomeruli of LPS-STZ (Fig. 1). Immunoreaction products with anti-E-selectin were not detected in kidneys of ICR (Supplementary 1), LPS-ICR or STZ-ICR while the products were identified in the whole renal parenchyma including glomeruli of LPS-STZ (Fig. 2). The Mac-1-positive cells were rarely identified in the whole renal parenchyma of ICR (Supplementary 1). The Mac-1-positive cells were identified in the whole renal parenchyma of LPS-ICR and STZ-ICR (Fig. 3).

### Immunostaining of FGF23 and ACE2

**Fig. 4:**
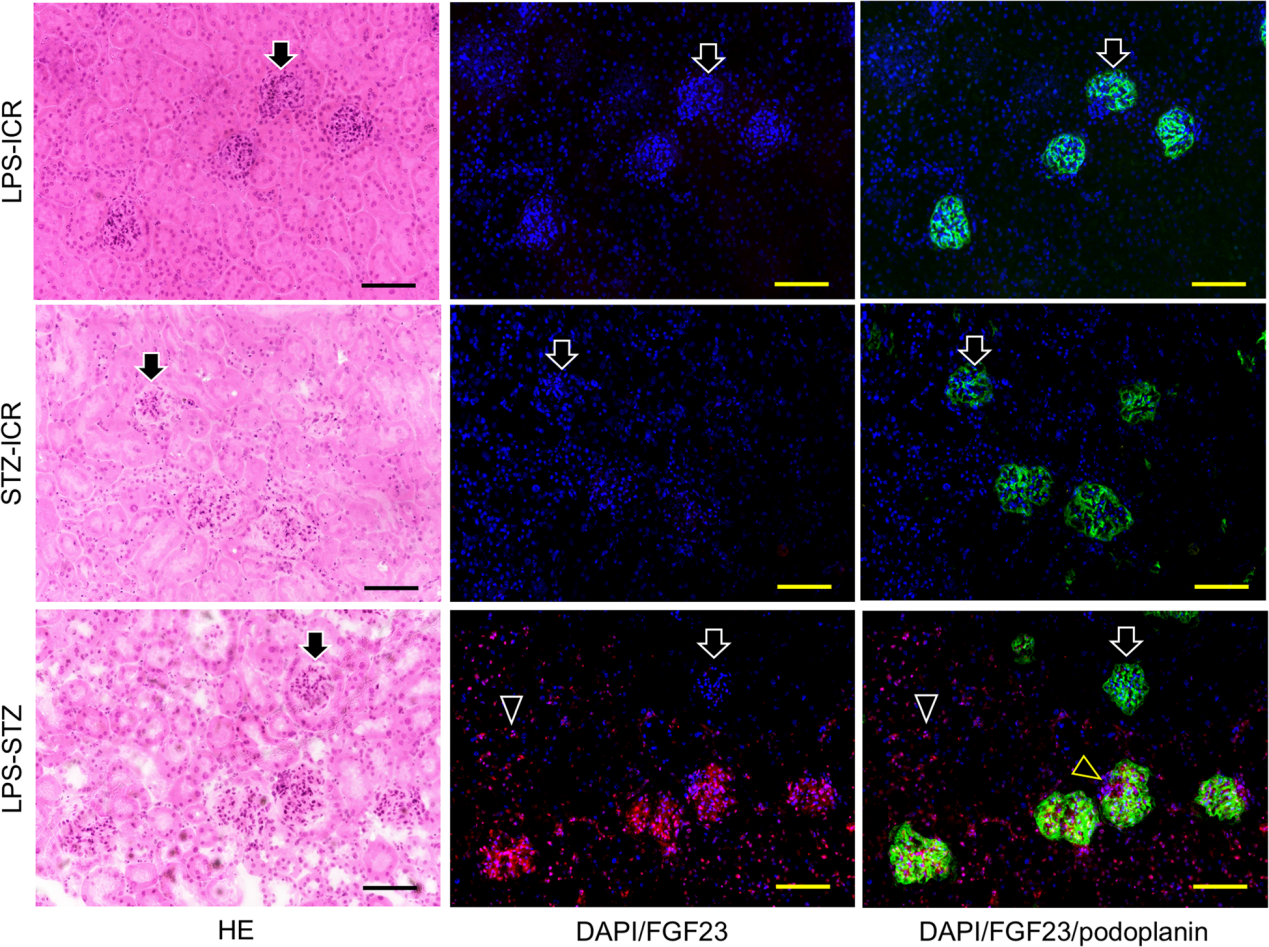
Immunostaining of FGF23 in the diabetic mouse kidney with Pg*-*LPS-induced nephropathy. Hematoxylin-Eosin staining (HE) (left column); immunostaining for FGF23 (red) (center column); and merged immunostaining for FGF23 and podoplanin (green) (right column), with DAPI staining of nuclei (blue). The glomerular epithelial cells were immunostained by anti-podoplanin to be able to discriminate glomeruli (arrows). Reaction with anti-FGF23 was not identified in LPS-ICR (top row) or in STZ-ICR (middle row) while the reaction was identified in the whole renal parenchyma (arrowheads) and glomeruli (yellow arrowhead) in LPS-STZ (bottom row). Bars: 100 μm

**Fig. 5:**
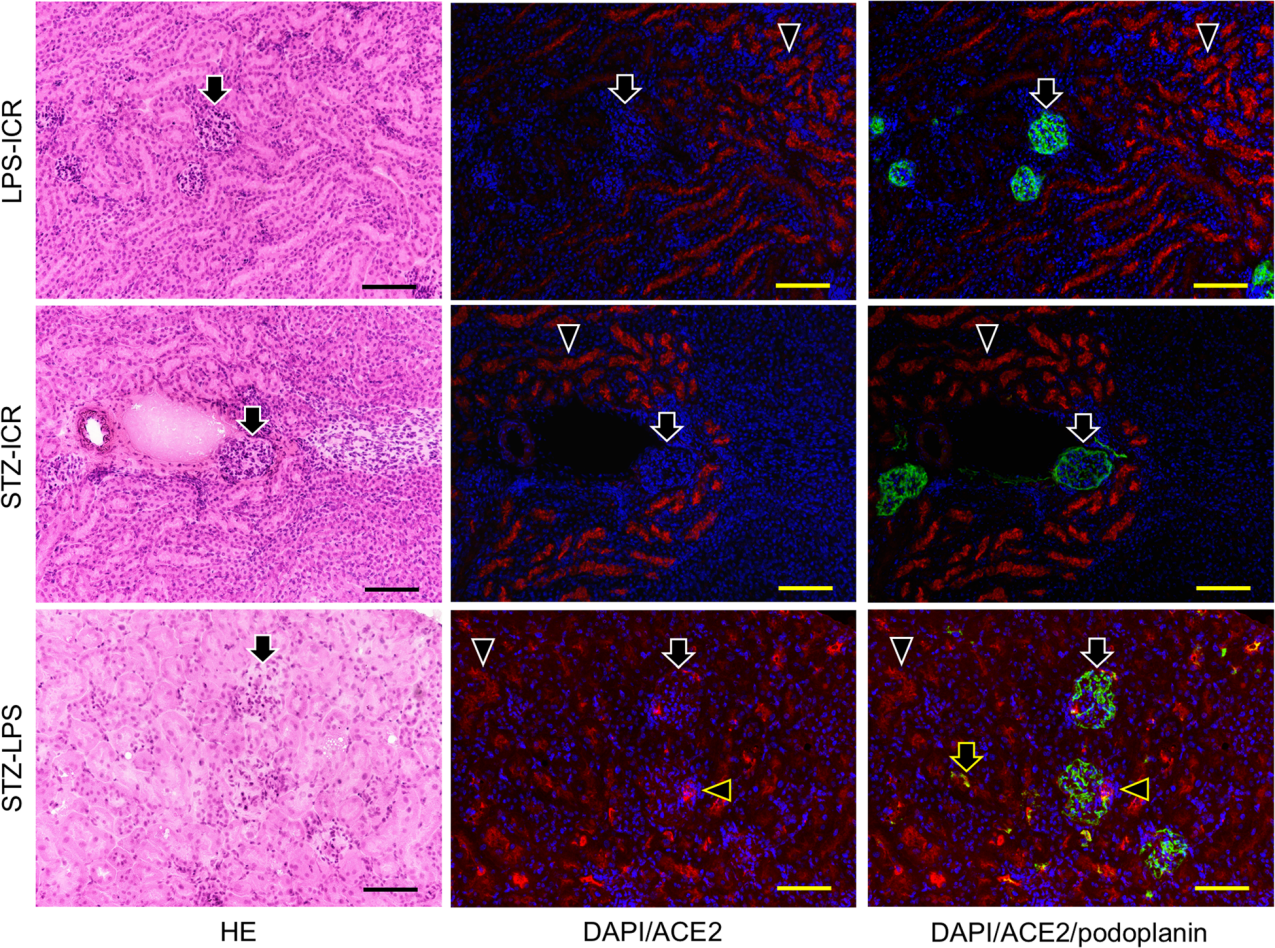
Immunostaining of ACE2 in the diabetic mouse kidney with Pg*-*LPS-induced nephropathy. Hematoxylin-Eosin staining (HE) (left column); immunostaining for ACE2 (red) (center column); and merged immunostaining for ACE2 and podoplanin (green) (right column), with DAPI staining of nuclei (blue). The glomerular epithelial cells are immunostained by anti-podoplanin to be able to discriminate glomeruli (arrows). Reaction with anti-ACE2 (arrowheads) was only observed in the proximal tubular cells with brush borders but not in any other region including distal tubules in LPS-ICR (top row) or in STZ-ICR (middle row) while the reaction was identified in proximal and distal tubules, and in glomeruli (yellow arrowheads) in LPS-STZ (bottom row). Podoplanin-positive macrophages were also identified in LPS-STZ (yellow arrow). Bars: 100 μm

Immunoreaction products with anti-FGF23 were not observed in kidneys of ICR (Supplementary 2), LPS-ICR or in STZ-ICR while the products were identified in the whole of the renal parenchyma including in glomeruli of LPS-STZ (Fig. 4). Immunoreaction products with anti-ACE2 were observed in proximal tubular cells with brush borders but not in any region including distal tubular cells in kidneys of ICR (Supplementary 2), LPS-ICR or STZ-ICR while the products were identified in proximal and distal tubules, and in glomeruli of LPS-STZ (Fig. 5).

### Quantitative analysis for immunostaining

The renal VCAM-1 and E-selectin positive area were significantly larger in LPS-STZ than in ICR, LPS-ICR, or STZ-ICR. The Mac-1 and podoplanin positive area were significantly larger in LPS-STZ than in ICR, LPS-ICR, or STZ-ICR, and the Mac-1 and podoplanin positive area were also significantly larger in STZ-ICR than in ICR, or LPS-ICR. The renal FGF23 and ACE2 positive area were significantly larger in LPS-STZ than in ICR, LPS-ICR or in STZ-ICR.

## Discussion

### Expression of leukocyte adhesion molecules in diabetic mouse kidney with *P. gingivalis* LPS-induced nephropathy

The expression of adhesion molecules increases in diabetic kidneys [30, 33]. An immunoglobulin superfamily member VCAM-1 binds to the integrins very late antigen-4/α4β7 on lymphocytes and monocytes, and provides leukocyte migration from the blood stream into tissue. The VCAM-1 expression is up-regulated in renal proximal tubules in several renal chronic diseases, and the renal proximal tubule is targeted in the renal infiltration of T cells and monocytes which are rarely found in normal kidneys [30–34]. A member of selectin family E-selectin which expresses at the early inflammatory stage binds to sialylated glycoproteins on leukocytes and promotes the leukocyte weak adhesion, rolling on vessel walls. The E-selectin is present on intertubular capillaries in glomerulonephritis but never in renal tubules [31, 35, 36]. In this study there were no vessels expressing VCAM-1 (Fig. 1) or E-selectin (Fig. 2) in kidneys of ICR, STZ-ICR and LPS-ICR while in LPS-STZ the expression of VCAM-1 was identified in the renal tubules and glomeruli (Fig. 1), and the expression of E-selectin was identified in the renal parenchyma including glomeruli (Fig. 2). These results suggest that Pg-LPS causes the renal inflammatory based on the overexpression of leukocyte adhesion molecule VCAM-1 and E-selectin in intertubular and glomerular capillaries of LPS-STZ. The overexpression of VCAM-1 in tubules of renal diseases has been reported [30–34]. The E-selectin is not expressed in tubules but it has been shown that renal intertubular capillaries express E-selectin in renal diseases, and that soluble E-selectin plays a role to promote glomerulonephritis [31, 35, 36]. The renal immunoreaction with anti-E-selectin in LPS-STZ may be ascribed to a soluble E-selectin diffused in renal parenchyma around glomeruli or to the expression of intertubular capillaries. It is thought that periodontitis induces the tubulitis because of the overexpression of VCAM-1 and E-selectin under diabetic conditions and promotes the nephropathy.

Macrophages, Th17, and lymphatic endothelial cells express podoplanin [43, 44]. Macrophages are distinguishable because size of Th17 is about the same as the diameter of the nucleus and the renal lymph vessels are much larger as described elsewhere [45]. The Mac-1 is a heterodimer integrin composed of the αM (CD11b) and β2(CD18) subunits and is abundantly expressed on monocyte/macrophages, and is critical for the adhesion and migration into the extracellular matrix [46, 47]. In this study Mac-1 positive cells were detected in kidneys of ICR, STZ-ICR and LPS-ICR at the similar levels while the distribution of Mac-1 and podoplanin-positive cells was remarkable in STZ-ICR compared to LPS-ICR, suggesting that diabetic conditions promote renal inflammatory events. The distribution of Mac-1-positive cells was remarkable in LPS-STZ compared to LPS-ICR or in STZ-ICR (Fig. 3). Since the overexpression of VCAM-1 and E-selectin was observed in glomeruli, tubules, and intertubular capillaries in LPS-STZ, it is thought that periodontitis provokes chronic inflammatory events by the renal monocyte-macrophage lineage infiltration under diabetic conditions.

### Expression of renal physiologically active substances in diabetic mouse kidneys with *P. gingivalis* LPS-induced nephropathy

The FGF23 directly targets proximal tubules to increase phosphate excretion by downregulating the cell surface expression of the sodium-dependent phosphate transporters in the proximal tubule. The FGF23 lowers serum phosphorus concentrations by the suppression of phosphorus reabsorption in proximal tubules and by active vitamin D reduction through 1α-hydroxylase suppression [37–39]. In this study FGF23 was not detected in kidneys of ICR, STZ-ICR and LPS-ICR while FGF23 was detectable in renal tubules and glomeruli of LPS-STZ (Fig. 4), suggesting that Pg-LPS promoted the accumulation of FGF23 in diabetic renal tubules. Generally, diabetic nephropathy reduces the ability to excrete phosphorus in renal tubules. Diabetic nephropathy leads to a compensatory increase in blood FGF23 levels and the renal accumulation of unmetabolized FGF23, resulting in hypertension and cardiovascular diseases [8, 48–51]. It may be postulated that periodontitis provokes renal tubulitis and accumulates unmetabolized FGF23 in the kidney under diabetic conditions, and that the increase of blood FGF23 in diabetic patients with periodontitis may contribute to provide a prediction of the nephropathy progression.

It has also been shown that proximal tubular epithelial cells in the brush border express ACE2 and the expression increase in diabetic kidneys and in hypertensive renal diseases [40, 41]. In this study ACE2 were only detected in the proximal tubular cells but not in any other region in kidneys of ICR, STZ-ICR and LPS-ICR while ACE2 was detected in proximal and distal tubules, and in glomeruli in the kidney of LPS-STZ (Fig. 5). It is thought that periodontitis may induce the overexpression of ACE2 in proximal and distal tubules to protect renovascular hypertension in inflamed glomeruli under diabetic conditions. Periodontitis in patients with diabetic nephropathy may require attention to increased renal ACE2 that is a critical SARS-CoV-2 entry factor [52].

**Fig. 6:**
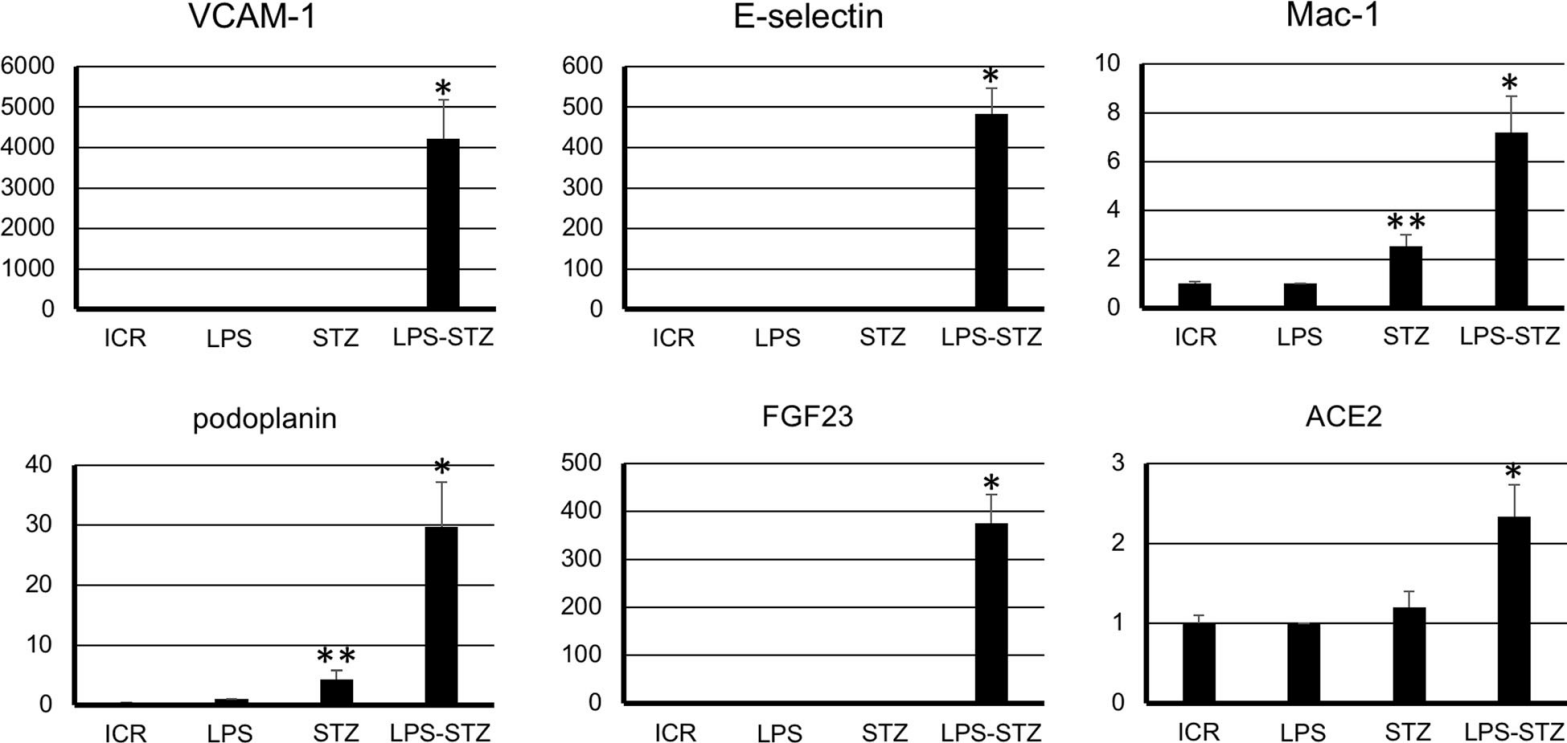
Quantitative analysis of the immunostaining of renal physiologically active molecules, leukocyte adhesion molecules, and leukocytes. Immunostained areas were measured in microscopic images by ImageJ. Relative expression area was expressed by arbitrary unit: Positive area of ICR (no treatment), STZ-ICR (STZ) and LPS-STZ / the area of LPS-ICR (LPS). The VCAM-1, E-selectin, Mac-1, podoplanin, FGF-23, and ACE2 positive area were significantly larger in LPS-STZ (*) than in ICR, LPS or STZ. The Mac-1 and podoplanin positive area were significantly larger in STZ (**) than in ICR, or LPS. *,**Significantly different by one-way ANOVA

**Fig. 7:**
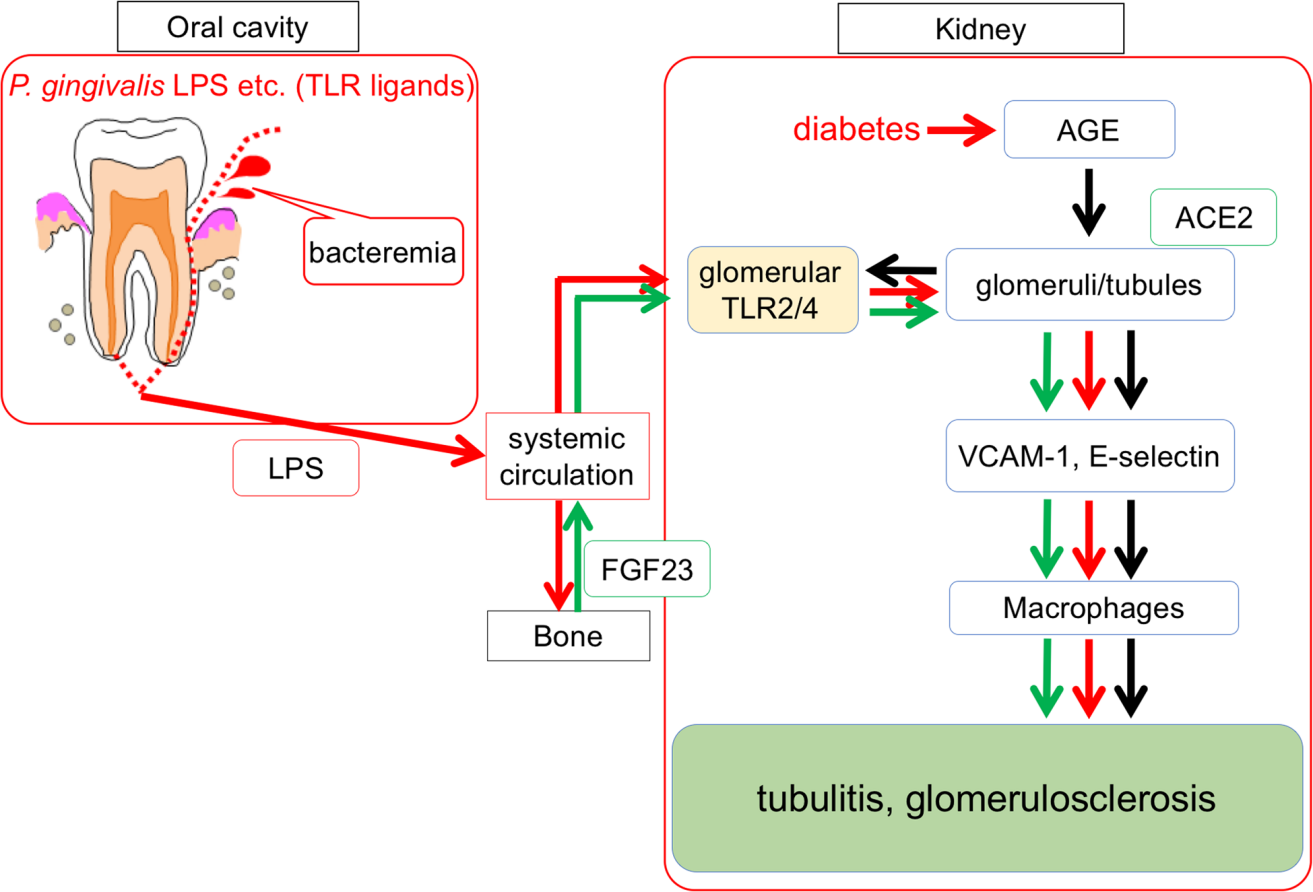
Prediction of complex etiology of *P. gingivalis* LPS-induced diabetic nephropathy. Severe periodontitis causes bacteremia and microorganisms of the oral cavity enter kidneys through the systemic circulation. AGE induces renal TLR expression, recognized by TLR2/4, and promotes the expression of cytokines and leukocyte adhesion molecule VCAM-1, and E-selectin. Inflammatory infiltration of Mac-1/podoplanin positive macrophages causes with overexpression of leukocyte adhesion molecules in *P. gingivalis* LPS-accumulated renal glomeruli, and simultaneously ACE2 overexpression and bone-derived FGF23 accumulation promote tubulitis and diabetic nephropathy with hypertensive renal diseases

In summary, the renal expression of leukocyte adhesion molecule VCAM-1 and E-selectin, the renal distribution of Mac-1/podoplanin-positive leukocytes, and the renal accumulation of unmetabolized physiologically active molecule FGF-23 and ACE2 were significantly larger in LPS-STZ than in ICR, LPS-ICR or STZ-ICR (Fig. 6). Normal inhabitants and LPS derived from intestinal bacteria like *Escherichia coli* enter the liver via the enterohepatic (portal) circulation and bacteria/harmful substances are sterilized there, so sepsis does not occur. However, oral bacteria enter the systemic circulation directly without passing through a detoxification organ such as the liver. It is the anatomical reason for the head and neck infections spread through the whole body and it can be easily predicted that oral bacteria enter the kidney via the systemic circulation [19–22]. The number of Mac-1 and podoplanin-positive cells was also significantly larger in STZ-ICR than LPS-ICR. It is well-established that severe periodontitis causes bacteremia and microorganisms of the oral cavity enter the systemic circulation, and that large amounts of LPS produced in *P. gingivalis* act as a risk factor in cardiovascular disorders [53–55]. It has been thought that AGE induces TLR expression in several somatic cells and that AGE is recognized by TLR as well as AGE receptor [6–8]. We recently reported that the glomerular endothelium of diabetic mice expresses TLR2 and TLR4, and that the TLR2/4-ligand *P. gingivalis* LPS causes glomerulosclerosis in diabetic mice with the accumulation of type 1 collagen and inflammatory cytokines in glomeruli [27–29]. Considering the present study, it was thought that Pg-LPS induced diabetic renal inflammation such as glomerulosclerosis and tubulitis with infiltration of Mac-1/podoplanin positive macrophages via glomerular overexpression of VCAM-1 and E-selectin, resulting in accumulation of both ACE2 and FGF23 which were unmetabolized with the inflammation-induced kidney damage under the diabetic condition (Fig. 7). As nephritis progresses, physiologically active substances and enzymes accumulate without being metabolized due to renal dysfunction. The compensatory productions to recover from dysfunction accelerate the accumulation in the kidneys and elevates the blood levels at terminal stages, and hyperphosphatemia simultaneously occurs because phosphorus cannot be excreted due to damage of renal tubules. Periodontal disease may contribute to the progression of stage in diabetic patients.

## Conclusion

It may be postulated that periodontitis provokes chronic renal inflammation by the renal overexpression of leukocyte adhesion molecules in diabetes patients with the renal accumulation of physiologically active substances.

## Supplementary Information

**Additional file 1 Supplementary 1.** Immunostaining of VCAM-1, E-selectin and Mac-1 in ICR. Hematoxylin-Eosin staining (HE) (left column); immunostaining (center column) for VCAM-1 (top row), E-selectin (middle row), and Mac-1 (red, bottom row); and merged immunostaining (right column) for VCAM-1/E-selectin/Mac-1 with podoplanin (green) and DAPI staining of nuclei (blue). The glomerular epithelial cells were immunostained by anti-podoplanin to be able to discriminate glomeruli (arrows). Reaction products were not identified for anti-VCAM-1 and anti-E-selectin; rarely identified for anti-Mac-1 (arrowheads). Bars: 100 μm.

**Additional file 2 Supplementary 2.** Immunostaining of FGF23 and ACE2 in ICR. Hematoxylin-Eosin staining (HE) (left column); immunostaining (center column) for FGF23 (top row) and ACE2 (bottom row); and merged immunostaining (right column) for FGF23/ACE2 with podoplanin (green) and DAPI staining of nuclei (blue). The glomerular epithelial cells were immunostained by anti-podoplanin to be able to discriminate glomeruli (arrows). Reaction products were not identified for anti-FGF23; identified for anti-ACE2 in the proximal tubules (red, arrowheads). Bars: 100 μm.

## Data Availability

The datasets during and/or analyzed during the current study available from the corresponding author on reasonable request.
