# Supplementary figures and images for: Immunohistochemical study for the expression of leukocyte adhesion molecules, and FGF23 and ACE2 in P. gingivalis LPS-induced diabetic nephropathy
Source: BMC Nephrol. 2021 Jan 6;22:3. doi: 10.1186/s12882-020-02203-y (PMC7786162; doi:10.1186/s12882-020-02203-y)

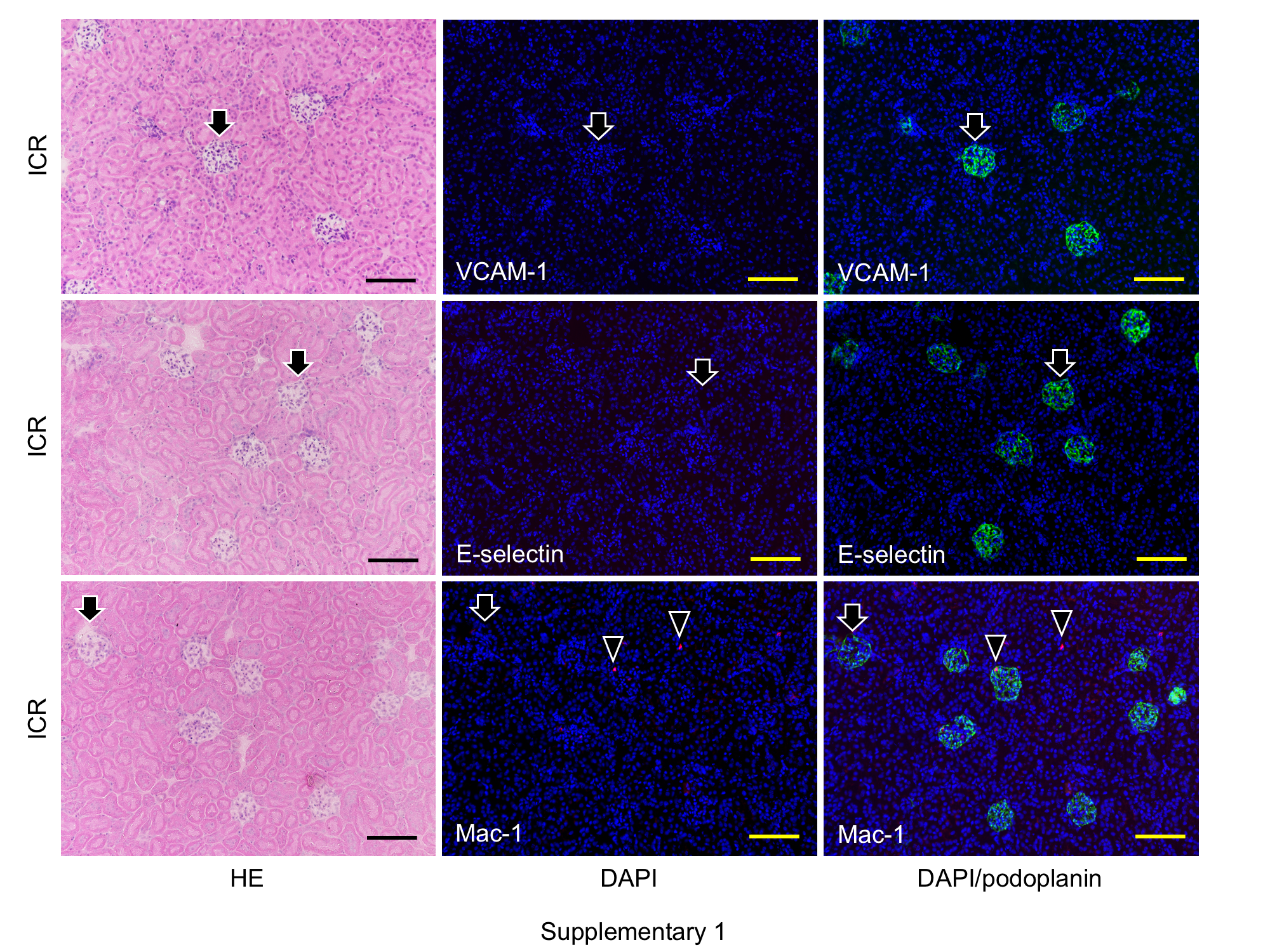

Supplement: Supplementary file 1 — Additional file 1 Supplementary 1. Immunostaining of VCAM-1, E-selectin and Mac-1 in ICR. Hematoxylin-Eosin staining (HE) (left column); immunostaining (center column) for VCAM-1 (top row), E-selectin (middle row), and Mac-1 (red, bottom row); and merged immunostaining (right column) for VCAM-1/E-selectin/Mac-1 with podoplanin (green) and DAPI staining of nuclei (blue). The glomerular epithelial cells were immunostained by anti-podoplanin to be able to discriminate glomeruli (arrows). Reaction products were not identified for anti-VCAM-1 and anti-E-selectin; rarely identified for anti-Mac-1 (arrowheads). Bars: 100 μm. [file 12882_2020_2203_MOESM1_ESM.tiff]

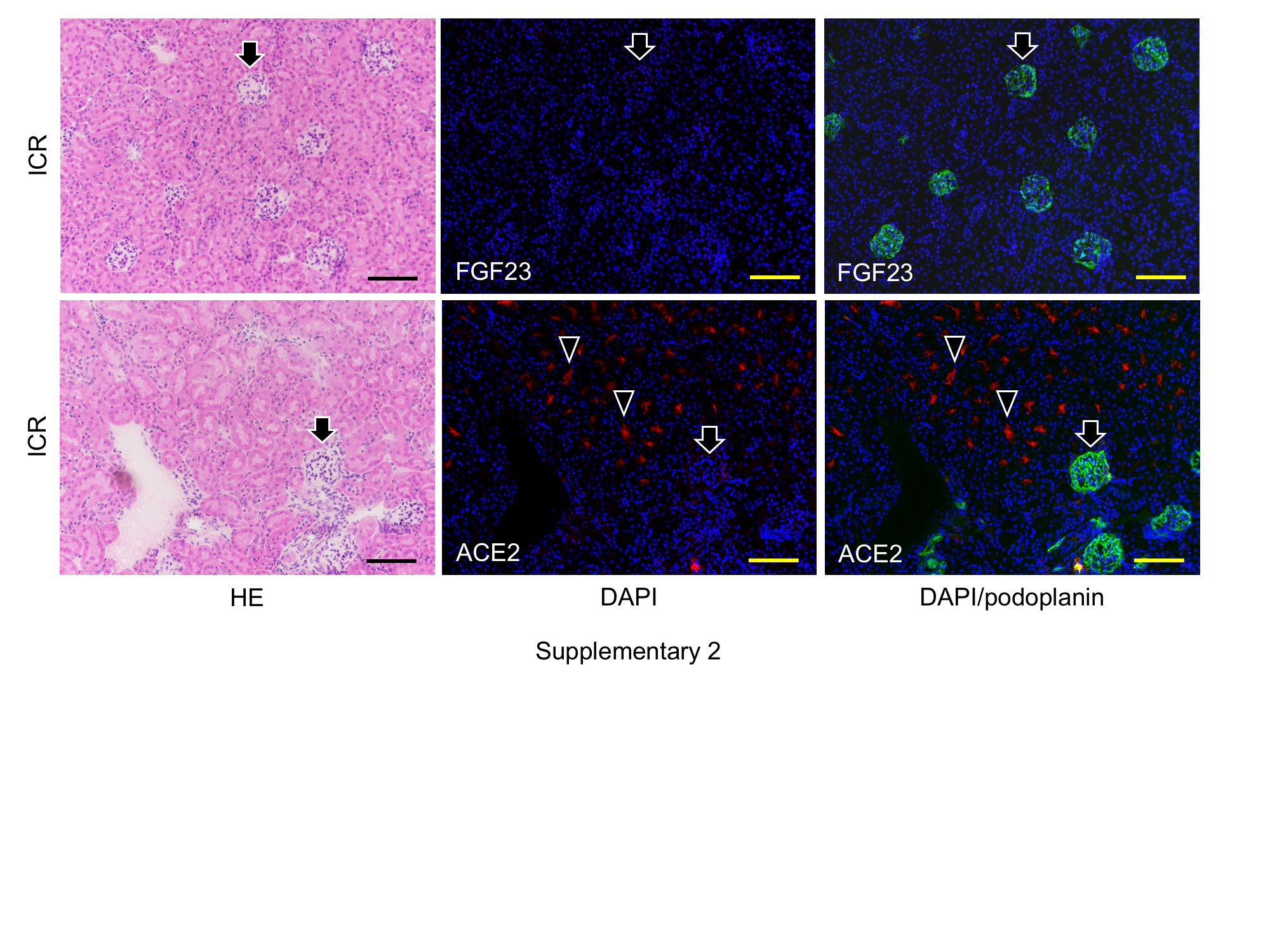

Supplement: Supplementary file 2 — Additional file 2 Supplementary 2. Immunostaining of FGF23 and ACE2 in ICR. Hematoxylin-Eosin staining (HE) (left column); immunostaining (center column) for FGF23 (top row) and ACE2 (bottom row); and merged immunostaining (right column) for FGF23/ACE2 with podoplanin (green) and DAPI staining of nuclei (blue). The glomerular epithelial cells were immunostained by anti-podoplanin to be able to discriminate glomeruli (arrows). Reaction products were not identified for anti-FGF23; identified for anti-ACE2 in the proximal tubules (red, arrowheads). Bars: 100 μm. [file 12882_2020_2203_MOESM2_ESM.tiff]
